# The vocal tract as a time machine: inferences about past speech and language from the anatomy of the speech organs

**DOI:** 10.1098/rstb.2020.0192

**Published:** 2021-05-10

**Authors:** Dan Dediu, Scott R. Moisik, W. A. Baetsen, Abel Marinus Bosman, Andrea L. Waters-Rist

**Affiliations:** ^1^ Laboratoire Dynamique De Langage (DDL) UMR 5596, Université Lumière Lyon 2, Lyon, France; ^2^ Division of Linguistics and Multilingual Studies, Nanyang Technological University, Singapore, Republic of Singapore; ^3^ RAAP Archeologisch Adviesbureau b.v., Leiden, The Netherlands; ^4^ DFG Center for Advanced Studies ‘Words, Bones, Genes, Tools: Tracking Linguistic, Cultural, and Biological Trajectories of the Human Past’, Eberhard Karls Universität Tübingen, Tübingen, Baden-Württemberg, Germany; ^5^ IDDS Groep b.v., Noordwijk, The Netherlands; ^6^ Department of Anthropology, The University of Western Ontario, London, Canada

**Keywords:** vocal tract, phonetics, osteology, language change

## Abstract

While speech and language do not fossilize, they still leave traces that can be extracted and interpreted. Here, we suggest that the shape of the hard structures of the vocal tract may also allow inferences about the speech of long-gone humans. These build on recent experimental and modelling studies, showing that there is extensive variation between individuals in the precise shape of the vocal tract, and that this variation affects speech and language. In particular, we show that detailed anatomical information concerning two components of the vocal tract (the lower jaw and the hard palate) can be extracted and digitized from the osteological remains of three historical populations from The Netherlands, and can be used to conduct three-dimensional biomechanical simulations of vowel production. We could recover the signatures of inter-individual variation between these vowels, in acoustics and articulation. While ‘proof-of-concept’, this study suggests that older and less well-preserved remains could be used to draw inferences about historic and prehistoric languages. Moreover, it forces us to clarify the meaning and use of the uniformitarian principle in linguistics, and to consider the wider context of language use, including the anatomy, physiology and cognition of the speakers.

This article is part of the theme issue ‘Reconstructing prehistoric languages’.

## 1. Introduction

Obviously, the dead cannot speak: we cannot listen to Shakespeare reading *A Midsummer Night's Dream*, we cannot elicit verb conjugations from Cicero, and we cannot even get Tutankhamun to say ‘aaah’ … The furthest we could get—which is incontestably a *tour de force*—was to CT-scan the mummified body of an Egyptian scribe and priest, Nesyamun, from about 3000 years ago, print a three-dimensional reconstruction of his vocal tract, and produce a creepy sounding [æ:::] [1]. That, and the various attempts to simulate Neanderthal vowels based on debatable reconstructions and assumptions, leading to endless debates about their capacity (or lack thereof) to articulate the ‘full modern’ vowel space, with a particular focus on [u] [2–6]. While we are far from a general consensus, language and speech go back at least to the origins of modern humans a few hundred thousands of years ago [7,8] but, most probably (given recent evidence), at least to the last common ancestor of modern humans and our closest evolutionary relatives, the Neanderthals and the Denisovans, about half a million years ago [9,10].

But our limits in what concerns the speech and language of long-gone people run deeper than this: there seems to a widespread assumption in linguistics that, on the one hand, there are precious few traces left by speech and language (writing goes back not more than a few thousand years) and, on the other, living (and attested) languages are very poor at retaining information about their earlier stages. Taken together, these seem to impose a ‘time horizon’ beyond which we cannot really know much [11], a time horizon that is usually placed at most 10 000 years ago, and rooted in the breakdown of the ‘standard’ historical linguistic comparative method of information recovery and inference ([12–15]; see also [16]). This breakdown results in the reluctance to connect established language families into larger (and, presumably, deeper) constructs such as ‘Nostratic’ [17,18], ‘Eurasiatic’ [19] or ‘Altaic’/’Transeurasian’ [20,21]. While this reluctance is clearly justified [11,17,22] by the daunting methodological and data availability issues, which make it very hard to distinguish biases and *a priori* stances from actual inferences from the data, it also makes it very hard to study these issues.

Nevertheless, there are intriguing hints that language might not be as bad at retaining information after all, especially when combined with external sources of evidence, such as (ancient) genetics [23–25] and archaeology [26], but accessing it requires the development and application of new methods, cross-disciplinary collaborations and, most importantly, the willingness to accept that false positives will inevitably be generated, but that the scientific process will weed (most of) them out. In this context, it is interesting to note that quantitative methods borrowed from evolutionary biology (especially Bayesian phylogenetics) have not only helped refine the internal structure of established language families (such as Indo-European, Austronesian, Uralic or Pama-Nyungan), but also, especially combined with external evidence, suggested ideas about their origins and spread [27–30], pushing the boundaries to 5000–8000 years ago. The same class of methods, however, has been used to explore even ‘deeper’ connections between languages, producing exciting but highly controversial results. Some of these results include: the apparent support for something akin to Eurasiatic (about 15 000 years ago) from phylogenetic methods applied to cognacy judgments [31] and to the alignments of actual Automated Similarity Judgment Program (ASJP; [32]) transcriptions [33]; the proposed Bayesian phylogenetic evidence for Transeurasian [20]; the very indirect finding that the stability of structural features of language might conserve continent-wide deep signals of shared ancestry and/or contact, suggesting connections between the language families of the Americas and north-eastern Eurasia going back approximately 15 000 years or so [34]; and, the bold claim that phonological systems might retain a signal of the modern human expansion from Africa some tens of thousands of years ago [35,36]. However, besides being currently unclear how reliable these findings are [22,37,38], they only push our knowledge back but a sliver of the half million years or so of human speech and language [9,10], and remain, in general, fairly abstract and ‘high level’. Can we do better?

Here we suggest that we might be able to infer fairly concrete information about past phonetics and phonologies, going back as far as the fossil record of the Neanderthals and other ‘archaic humans’ (half a million—or even more—years ago). To do so, we will use the links between aspects of the vocal tract anatomy, and the articulation and acoustics of speech which are uncovered by recent investigations into the patterns of inter-individual variation in the anatomy of the speech organs. Besides allowing us to make informed guesses about Neanderthals lacking labiodentals and the persistence of clicks in sub-equatorial Africa, this approach also questions the indiscriminate application of a strong uniformitarian principle to speech and language, arguing instead for a much more nuanced inferential framework that takes into account the wider context of language, which includes, among others, the physical environment and human biology [39,40].

## 2. Variation everywhere

Due to space constraints and the specific focus of this paper, we will only briefly summarize here points discussed at length in other publications (e.g. [39–41]). Recent advances in several scientific fields (including medicine, human genetics, anthropology and psychology), the availability of large computer-readable databases, the democratization of computing power, data analysis and statistics, and wider changes in how society at large, and science in particular, sees variation, have allowed a renewed interest in understanding how people vary, how this inter-individual variation is patterned, and how it relates to universal characteristics. What we realize is that, on a massive foundation of sharedness, individuals vary in subtle ways at all levels of study: from the molecular [42,43], to the anatomical and physiological [44], and to the psychological and cognitive [45]. This variation is mostly small and quantitative, and results in a wide range of ‘normality’ that grades into the ‘pathological’—here we are interested in this normal range of variation, which is not distributed at random between individuals, but is intricately patterned. Thus, on the one hand, any given individual belongs to multiple (overlapping or nested) groups, and groups differ in myriad continuous, statistical and multivariate ways (as opposed to ‘crisp’, deterministic, categorical differences driven by one or a few characteristics, as usually claimed by racist ideologies). This patterning is rooted in our complex but relatively recent evolutionary and demographic history [46–48]: while we are much more uniform than other species (for example, there is less genetic diversity in the 8 billion people spread across the world than in a few hundred highly geographically circumscribed chimpanzees; [49]), we do vary, with most of this variation distributed between individuals (approx. 80%), and not between groups either within (approx. 10%) or between continents (approx. 10%) [46,50]. Yet, this variation is informative enough (especially when aggregated among many genetic loci and characteristics) to recover individual origins, geographic patterns of human dispersals and migrations, and past demographic events [48,51]. This variation decreases with distance from Africa, is distributed as continuous and overlapping gradients across many variables, with very few (if any) sharp boundaries—often referred to as ‘clinal variation’ [46,47,52]. Clinal variation underscores the unity of humankind, and a proper understanding of its patterns and processes represents one of the most powerful scientific arguments against racism, sexism and other forms of discrimination [48,53–56].

Language in general, and the vocal tract in particular, are far from being exceptions, despite decades of focusing on (absolute) universals and denying, or, at best, dismissing and trivializing variation as mere irrelevant ‘noise’ to be removed from the analysis and ignored from theorizing. Of course, this was not a blanket stance, with aspects of variation being actively studied in, for example, dialectology [57], sociolinguistics [58] and phonetics [59–64], but it did result in the marginalization of such inquiries and limited the appreciation of how widespread variation is, and of how powerful an explanatory factor it may be [39,41]. However, focusing on phonetics and phonology alone, the last two decades have witnessed the accumulation of data concerning the type and patterns of inter-individual variation in the production and perception of speech, as well as a heightened interest in the theoretical implications for phonology, sound change and typological diversity [41,65].

Of particular interest here is variation in the anatomy of the vocal tract structures, especially in those structures that (i) have a higher chance of preservation in the osteological and fossil record, or (ii) whose particularities can be inferred from ancient DNA, and their effects on phonetics and phonology. While our understanding of the genetic and developmental underpinnings of the vocal tract is still in its infancy, being primarily based on pathologies with a genetic component (see, for example, the information available in *OMIM*, https://omim.org/), there are several candidate genes and mechanisms known, especially concerning the teeth [66,67] and the hard palate [68,69], but also the skull and the face in general (see, for example, *FaceBase*; https://www.facebase.org/). These sources of information are rich enough to even allow some inferences about the evolution of the position of the larynx and the structure of the face from methylation patterns in ancient DNA extracted from archaic human fossils [70]. However, we are far from being able to reliably reconstruct the details of normal variation in vocal tract anatomy from the (epi)genetic variants ascertained from the remains of a given individual, which means that, at least for now, we should focus instead on the osteological and fossil record, which has the advantage that the relevant anatomical details are sometimes preserved well enough, but is affected by the twin disadvantages of very small (and geographically and historically skewed) sample sizes, and the lack of soft tissue preservation (with a few notable exceptions involving special taphonomic conditions such as anaerobic bogs, permafrost or ice; [71,72]). It is also important to recognize the potential influence of environmental factors on the physiology and anatomy of the vocal tract. The desiccation of the vocal folds, and its subsequent phonatory consequences, in environments with low air humidity [73,74] is an example of the first type, while the sexually dimorphic and geographically patterned variation in the nasal cavity [75–77], which seems partly explained by the need to warm and humidify the in-breathed air in cold and dry climates [76,78–80], with possible consequences for nasalization, is an example of the second type.

Before discussing the type of vocal tract data that can be recovered from this record, and the kind of inferences for speech and language that can be made, we briefly review a few studies linking variation in details of vocal tract anatomy to phonetics and phonology using a multitude of methodologies, including experimental designs with living people, computational modelling and phylogenetic inferences.

## 3. From details of the vocal tract to phonetic and phonological diversity

To begin, there are well-known claims that the position of the larynx within the throat can be inferred from traces in the archaeological record, most notably the shape of the base of the cranium and characteristics of the hyoid bone, and that this positioning might allow us to say something about the capacity of ancient humans to produce (or not) the full modern vowel space [2,3,5,6,9]. However, despite claims to the contrary [81,82], it is far from clear how reliable such reconstructions are (even when adding inferences from ancient epigenomes; [70]), but, more importantly, it is not obvious what articulatory, acoustic and linguistic effects different positions of the larynx would have. The original claims [6] that Neanderthals had a much higher larynx than modern humans and that this precluded them from producing the full spectrum of vowels found in the currently spoken languages, did not age well. Newer models of speech articulation seem to suggest that the vowel space produced with a higher larynx is not terribly limited thanks to active compensation by the other articulators [2–4], that the rest position of the larynx is not very relevant given its wide dynamic range [83,84], and that the Neanderthal hyoid bone was, in fact, anatomically and biomechanically extremely similar to the modern human one [85]. Finally, despite the importance of the peripheral vowels, and the fact that actual speech productions are spread across the potential (acoustic and articulatory) vowel space relatively independently of the described phonological system of the language, few modern languages use the full extent of this potential space to convey phonological distinctions.

As we will detail below, the hard structures of the vocal tract have by far the highest chances of preservation in the osteological and fossil record, suggesting that we should focus on the hard palate, the jaw and the teeth. There is tremendous inter-individual morphological variation in all these structures, and some aspects of this variation are patterned, in a continuous, statistical and multivariate manner, also between groups [39,41]. We will briefly review a few studies, using a variety of methods and data, showing how normal variation in these structures affects speech.

There are experimental indications that the midsagittal shape of the hard palate—between ‘domed’ and ‘flat’—influences token-to-token variability, with speakers with ‘flatter’ palates showing less articulatory variability [61,86]. Computer models combining a realistic geometric model of the vocal tract (VocalTractLab 2.1; https://www.vocaltractlab.de/; [87]) controlled by a neural network, which repeatedly learns and transmits vowels across multiple generations [88], show that details of the midsagittal shape of the hard palate, as quantified using Bézier curves [89], do affect the articulation and acoustic properties of vowels. Importantly, the acoustic effects survive the active compensation by the free articulators (the tongue, jaw and lips) and, despite being very weak in any particular generation, are amplified by repeated learning across generations, to the level of the acoustic variation observed within actual languages [88].

Zooming in on a specific substructure of the hard palate, the alveolar ridge (a shelf-like structure behind the upper incisors), biomechanical models using ArtiSynth [90] show that its shape and size affect the effort required for the articulation of click consonants as well as their acoustic properties [91]. Specifically, the alveolar ridge shape that is statistically most often found among the native speakers of ‘click languages’ in southern Africa (i.e. a ‘small’ or ‘absent’ alveolar ridge) seems to reduce the effort needed and may simultaneously enhance the acoustics of clicks. There is also experimental evidence from a large, ethnically diverse sample, using structural, sustained articulation and real-time magnetic resonance imaging (MRI), complemented with intra-oral optical three-dimensional scans and acoustic recordings [41], showing that the strategy used to articulate the North American English ‘r’ is influenced by the anatomy of the anterior vocal tract, including the hard palate, the alveolar ridge and the lower jaw. While practically indistinguishable from an acoustic point of view, these different articulations nevertheless influence the articulation and acoustics of other neighbouring sounds [92,93], thus potentially influencing sound change.

The last study to be mentioned here [94] combines biomechanical modelling, large-scale cross-linguistic statistical analyses, detailed case studies, and Bayesian phylogenetic analyses of the Indo-European family. It shows that the type of bite (‘overjet’/’overbite’ versus ‘edge-to-edge’) most common in a population influences the probability that the language(s) spoken by that population will have labiodental sounds (such as ‘f’ and ‘v’) in their sound system. Importantly for us here, the type of bite is strongly influenced by post-developmental factors, especially the diet, with hunter-gatherer populations predominantly showing an ‘edge-to-edge’ bite, while those practicing agriculture predominantly having ‘overjet’/’overbite’ [94]. This influence is mediated by at least two processes having to do with the mechanical properties of food: tougher foods promote tooth erosion and movement [95–97], but also affect the growth of the lower jaw [98].

Taken together (see [39,41] for more comprehensive reviews and discussions), these studies suggest that (a) there is extensive normal inter-individual and, in some cases, even between-group variation in the hard structures of the vocal tract (e.g. hard palate, teeth, lower jaw); (b) this variation is mostly continuous, statistical and gradient in nature, but it (c) sometimes does influence the articulation and/or the acoustics of speech sounds, producing (d) (usually very) weak effects at the individual level (so-called ‘biases’) that nevertheless can be (e) amplified by the repeated use and transmission of language to result in unmistakable differences between dialects and languages in their phonetics and phonology. While this causal chain is very complex, influenced by many other factors (e.g. linguistic, cultural, historical, demographic, environmental) and composed of individually subtle links, it does seem to work, at least in some cases. This raises the hope that we may be able to infer something about past languages by combining such findings with information about vocal tract structures preserved in the osteological and fossil record—to which we now turn.

## 4. Aspects of the vocal tract that can be recovered from the osteological and fossil record

In what concerns the osteological and fossil traces of speech and language, a lot of attention has been given to the hyoid bone, the ear structures housed in the temporal bone, the openings in bones through which nerves can pass (i.e. foramina, canals), and to endocasts (fossilized brain traces). To date, we have fossilized hyoids from australopithecines [99], *Homo heidelbergensis* and Neanderthals ([85,100,101]; the finding originally identified as a *Homo erectus* hyoid [102] has been reinterpreted as a fragment of a vertebra [103]), and it seems that, while the *Australopithecus* hyoid is clearly different from the modern human one, the Neanderthal and pre-Neanderthal ones are very similar to our own [9,104]. The shape of the ear structures can be reconstructed using computed tomography (CT) scans, and ear ossicles are sometimes directly preserved [105–108], allowing inferences about the audition of long-gone humans: from these, it seems that, functionally, the hearing of Neanderthals was very similar to that of modern humans and clearly different from that of chimpanzees [9,10]. Unfortunately, brain endocasts [109,110] and the size of the hypoglossal canal [111,112] seem to currently offer rather limited and unclear evidence concerning speech and language.

**Figure 1.**
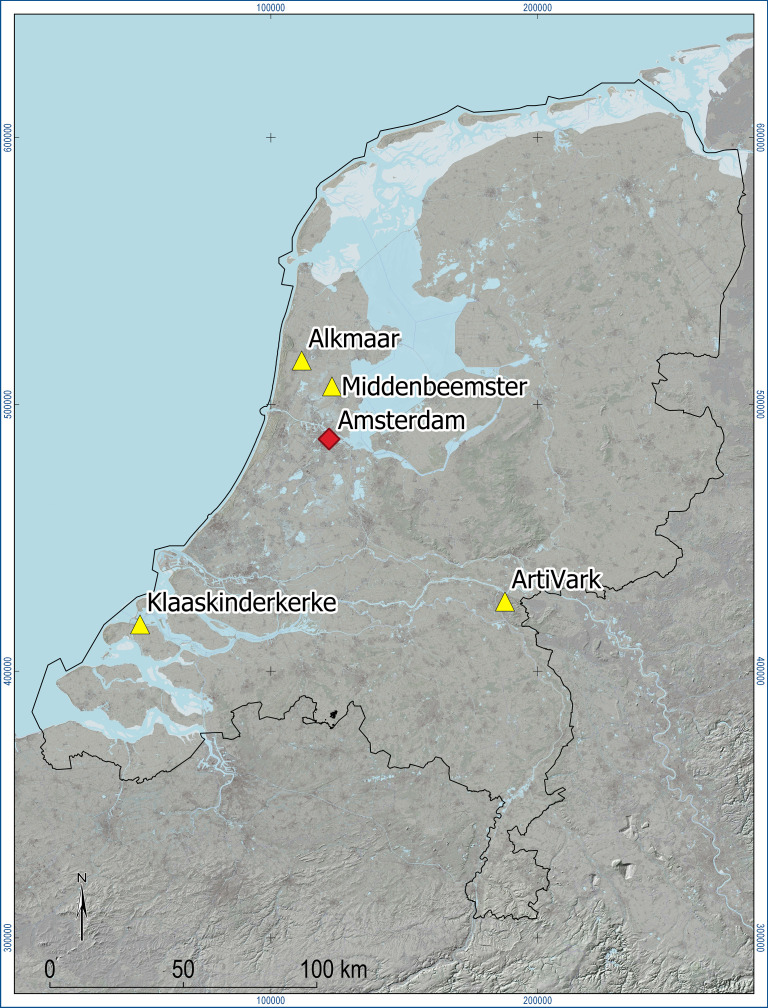
The map of The Netherlands showing the locations of the four samples (yellow triangles) as well as the location of Amsterdam (red diamond) for orientation. *ArtiVarK* is shown as located in Nijmegen. Map generated using QGIS, version 3.10.3-A Coruña.

However, the evidence briefly reviewed above seems to suggest that we might want to also focus on other components of the vocal tract, particularly on those with a bony component, increasing their chances of surviving the taphonomic processes. Evidence suggests that while the hard palate is a rather fragile structure, it is often quite complete when the cranium is not too fragmented, while the lower jaw survives rather well [113,114]. In fact, in order to test the feasibility of extracting information about the hard palate and the lower jaw from osteological remains, in 2015–2016 we conducted an exploratory study of well-understood collections of historic human skeletons (coming from different cemeteries). The overarching goals were to (a) digitize the structures of interest in order to (b) compare the historical samples to each other and with a modern sample, so that we can (c) ascertain the feasibility of the methods, (d) draw conclusions about temporal changes in vocal tract anatomy and, possibly, (e) their effects on speech and language. The project was a collaboration between D Dediu and SR Moisik (then at the Max Planck Institute for Psycholinguistics in Nijmegen, The Netherlands) and AL Waters-Rist, WA Baetsen and AM Bosman (then at the Laboratory for Human Osteoarchaeology, Faculty of Archaeology, Leiden University, also in The Netherlands), as part of the larger *G[ɜ]bils* (Genetic biases in language and speech) project, funded by The Dutch Research Council (NWO). Following a careful assessment, considering data availability, quality, sample size and meta-information, we decided to use one contemporary and three historical samples, all collected in The Netherlands (figure 1): *Alkmaar* (1484–1574 CE), *Klaaskinderkerke* (thirteenth to sixteenth centuries CE), *Middenbeemster* (1829–1866 CE), and part of the contemporary *ArtiVarK* sample (2014–2015).

*Alkmaar* (*Paardenmarkt*) is the oldest sample; it was part of a monastic cemetery in present-day Alkmaar in Noord Holland, in use between 1484 and 1574 CE. *Klaaskinderkerke* is a cemetery belonging to a *verdronken dorp* (lit. ‘drowned village’, or ‘sunken village’) on the island of Schouwen-Duiveland in the province of Zeeland, the remains there dating between the thirteenth and sixteenth centuries CE. *Middenbeemster* is a Protestant church cemetery in Noord Holland, located on a polder (or reclaimed lake), the Beemster, the first in The Netherlands to be dried by building dykes and pumping out the water using windmills; the remains are firmly dated between 1829 and 1866 CE. For more information about these historical samples, see previous work [115–117]. Finally, the *ArtiVarK* sample was collected between 2014 and 2015 in Nijmegen, and contains, of relevance here (see [41] for details), structural MRI and intra-oral three-dimensional optical scans of approximately 90 participants; for this study we used a subset of 34 (16 male; 18 female) contemporary Dutch individuals coming from across the whole country. See electronic supplementary material, table S1 for the list of included individuals.

For the hard palate (see [115] for details), we included 22 individuals from the *Klaaskinderkerke* sample (6 male, 11 probable male, and 5 probable female; 2 young adults, 6 young-middle adults, 10 middle and 4 middle-old adults—see below for details about the age categories), and 38 individuals from the *Middenbeemster* sample (17 male, 2 probable male, 3 probable female and 16 female; 22 young adults and 16 middle adults). For the lower jaw (see [116] for details), we included 37 individuals from the *Alkmaar* sample (6 male, 8 probable male, 5 female and 18 probable female; 20 young adults and 17 middle adults), and 51 individuals from the *Middenbeemster* sample (18 male, 10 probable male, 11 female and 12 probable female; 31 young adults and 20 middle adults). Note that the estimation of sex and age-at-death from osteological remains are methodologically complex, often difficult to perform due to damage resulting from taphonomy, and their results must be taken as broad probability estimates [118–120]. Therefore, we decided to split our individuals (all adults) into two broad groups: ‘young adults' (roughly 18–35 years old at death) and ‘middle adults’ (roughly 36–60 years old at death); note that the age ranges for the *Klaaskinderkerke* sample carry a greater uncertainty than the other collections, because of a general lack of postcranial remains. Likewise, we assign sex with certainty (male or female) whenever possible, but we also classified some individuals as ‘probably’ of one sex or the other. Note that, for the *Klaaskinderkerke* sample, a second, independent assignment of sex agrees with our own for all specimens except one (not used in the simulations conducted here).

The data acquisition and processing protocol was similar for the two structures: following a general osteological analysis, meta-data registration, and the application of various inclusion and exclusion criteria (good or excellent preservation, completeness, adult age, the absence of severe pathologies and abnormalities, and the absence of periodontal disease and ante-mortem tooth loss), the selected individuals were assessed for sex and their age-at-death was estimated. Next, the relevant structures were digitized using a NextEngine™ 3D Desktop Scanner Ultra HD 2020i (NextEngine, Inc., Santa Monica, CA), which is widely used in physical anthropology, palaeoanthropology and other fields. This device allows the acquisition of high-quality, high-resolution three-dimensional models of solid objects using multiple laser beams (for details, see [115,116]). This resulted in a set of three-dimensional meshes (post-processed in ScanStudio™ and MeshLab; [121]), one per individual and structure, on which landmarks (fixed, anatomically clearly defined features that are homologous between individuals) and semilandmarks (or ‘sliding landmarks’, i.e. a set of variable or ‘mobile’ points used to discretize a curve) were placed using Landmark Editor [122]: there are 27 landmarks for the lower jaw in the historical samples, 6 in the contemporaneous *ArtiVarK* sample (a subset of the 27, due to differing methodologies), and 44 for the hard palate (see electronic supplementary material, tables S2–S5 for a full list including the sliding semilandmarks; [115], tables 8 and 9, and figure 31 and [117], tables 2 and 3 for details; and electronic supplementary material, figure S8 for their placement). These allowed us to quantitatively compare the samples and individuals using classic and geometric morphometric [123] methods which allow the principled separation of variation in shape from variation in size.

In a nutshell (see [115] for full details), for the shape of the hard palate we found that our samples showed a large overlap: the variation within both samples is greater than that between them. This is to be expected, since the populations were not significantly separated temporally, geographically or linguistically (Middle Dutch versus modern Dutch; see [115,124–127]). The small amount of variation we found is best characterized by subtle differences in the height and shape (‘U’-shaped versus ‘V’-shaped) of the maxillary dental arch, as well as the flexion of the basicranial angle, the relative width of the nasal aperture and the degree of orthognathy. The only significant difference in average shape was found between the male group from *Klaaskinderkerke* and the female group from *Middenbeemster*. Size differences were found to be statistically significant between the two samples, with *Klaaskinderkerke* being slightly larger than *Middenbeemster*. The causes for these differences probably stem from developments during the respective periods when these people lived, ranging from climatic (temperatures during the Little Ice Age) to sociocultural (Industrial Revolution). For the lower jaw (see [116,117] for details), we found that differences between the two historic samples (*Alkmaar* and *Middenbeemster*) were dominated by size, with the male individuals from the older site (*Alkmaar*) having the largest mandibles on average, and the female individuals from *Middenbeemster* having the smallest mandibles. Moreover, the magnitude of sexual dimorphism seems to differ between the two sites, with a lower amount of sexual dimorphism present in the *Alkmaar* sample. The results are possibly linked to a softening of the diet that occurred between these time periods, although confounding factors such as sampling bias, life history, and shared population history could not be accurately accounted for.

There are several methods of palaeodietary reconstruction: while the faunal and floral remains at an archaeological site indicate the available foods, the analysis of various aspects of human bones and teeth indicate what people actually ate. The stable isotope ratios in bones and teeth paint a broad picture of the types of plants (C3 versus C4 photosynthetic pathway) and animals (herbivores versus carnivores, marine versus terrestrial) that were consumed [128], while dental calculus often contains masticated food debris, plant microfossils, protein biomolecules, and plant and animal DNA [129,130]. Such research has been successfully applied to both recent and ancient remains, including Neandertals and early modern humans [131,132], and may provide information about dietary variables that affect vocal tract anatomy. The macro- and microscopic analysis of dental wear can be useful for inferring how ‘hard’ or ‘soft’ the diet was [133,134]. Several of these methods have been applied to the three historical samples used here [135–137], but more research and better data integration are needed before we can study if (and how) dietary differences might have affected vocal tract anatomy. With the currently available data, it seems there were no major differences in dietary ‘softness’ among the archaeological populations, nor the modern *ArtiVarK* sample, suggesting that all groups had diets requiring broadly similar masticatory forces (but we cannot rule out that this is an artifact of the poor landmark coverage in the modern sample), concordant to the historical peasant staples of wheat or rye bread, dairy products (cheese, butter and milk), root vegetables, with smaller amounts of fish, and even more so, meat.

While still preliminary, these findings do show that (i) we can recover and quantitatively analyse data pertaining to the vocal tract from relatively old human remains, and (ii) that there are quantifiable differences between individuals, locations and historical periods that may be relevant for speech.

## 5. Getting the past to ‘speak’: simulating the articulation of vowels using medieval hard palates

**Figure 2.**
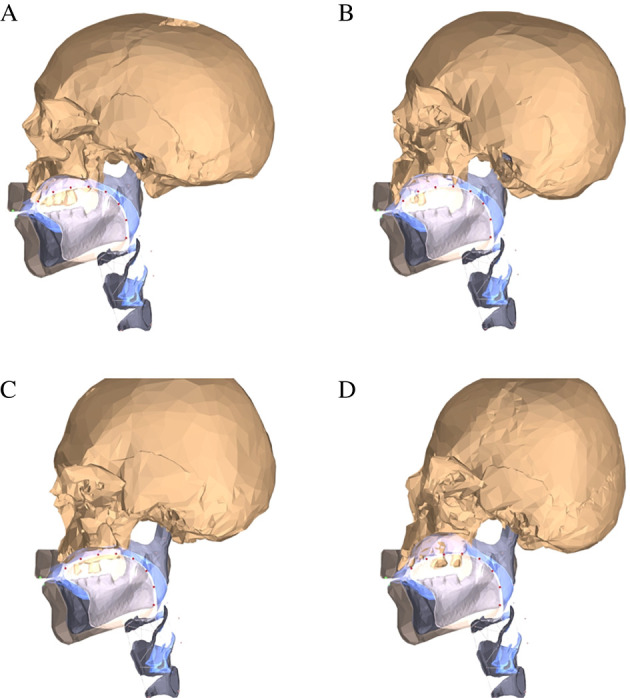
Midsagittal views (from the left) of the four reconstructed vocal tracts (identified as A–D; see the electronic supplementary materials for details). The airways are shown in blue, the associated skull samples are in beige and the large capital black letters are the samples. Sample access granted by the Stichting Cultureel Erfgoed Zeeland. Digitized image dissemination for educational purposes only. Crania models created by author W.A.B.; for access to the actual three-dimensional models of the crania, contact author W.A.B. (Online version in colour.)

Here we push this research programme an inch further, by making a subset of medieval hard palate samples ‘speak’. More precisely (figure 2; full details about the methods and results are available in the electronic supplementary materials ‘Modelling the biomechanics and acoustics of reconstructed vocal tracts: methods and full results', figures S1–S7 and Videos), we selected four individuals (two males, one probable male, one probable female; one young-middle adult, two middle adults, one middle-old adult) for which very complete skeletal geometry is available, and we used a biomechanical model of the vocal tract (ArtiSynth; [90]) to reconstruct, as accurately as possible, the way these individuals would have articulated the six vowels [i] (as in the North American English ‘heat’), [e] (as in ‘hate’), [æ] (as in ‘hat’), [ɑ] (as in ‘hot’), [o] (as in ‘hotel’) and [u] (as in ‘hoot’; figure 2; electronic supplementary material, figure S2 and Videos). What we found is that there are differences in the acoustics of the vowels between these individuals (electronic supplementary material, figures S4–S7), some of them rather dramatic (one case being that the vowel [e] of one individual is acoustically close to the [i] of the others). While our models are rather simplistic and, crucially, do not implement articulatory compensation or acoustic-auditory-based targeting, the differences we found are real in the sense that they would exist in the speech output of these individuals if they could maintain exactly the same articulatory structure and posture (given their individual hard palate and dentition); they are components of our organic voice quality [138,139]. Thus, the individuals must overcome these potential acoustic differences in their speech, through articulatory compensation, in order to achieve reasonably well the intended auditory vowel targets or risk being misunderstood.

Our own previous work [88], using a different type of model of the vocal tract that does allow for articulatory compensation of differences in the midsagittal shape of the hard palate through the use of the free articulators (mainly the tongue, the lips and the lower jaw), shows that even if this compensation is highly effective, it nevertheless fails to completely erase the acoustic ‘signature’ of inter-individual anatomical variation. This ‘attenuated’ acoustic signature is very small but present and, perhaps surprisingly, is sometimes amplified by the repeated use and transmission of language in populations composed of individuals with a similar anatomy [39,88,91].

All in all, we hope to have shown that (i) information about vocal tract structures can be successfully extracted from the remains of long-gone people, (ii) that it can be used in qualitative, quantitative and modelling investigations into (iii) the patterning of inter-individual (and, possibly, inter-group) variation with (iv) consequences for speech and language.

## 6. Discussion and conclusion

While the aforementioned data have only ‘scratched the surface’, we do hope to have shown that there is huge potential in such an approach, which uses traces of vocal tract anatomy from past people, combined with results from computer models and experiments in contemporaneous individuals, to make informed inferences about long-gone languages. What we have tried to show here concretely is that there is a wealth of information about specific ‘hard’ components of the vocal tract (the lower jaw and the hard palate) in the osteological record, that this information can be extracted and quantified in a rigorous manner, and that it can be used not only to compare individuals and groups across time, but also to simulate how these would have affected the production of vowels. Naturally, these first steps can be extended in time, space and linguistic coverage.

Temporally, we have focused here on relatively recent historical populations from well-understood contexts (medieval and post-medieval northwestern Europe) for pragmatic (reliable contextual information, good preservation, access to digitization technology, osteological expertise) and theoretical reasons (controversies about sexing using the lower jaw, changes in food and nutrition, sound changes in the history of Dutch). But our experience clearly suggests that this can be extended back in time for as far as there are well-preserved components of the vocal tract in the fossil record, emphatically preceding the emergence of anatomically modern humans a few hundred thousand years ago [140].

Geographically, there is nothing special about northwestern Europe besides the fact that it is historically rather well understood and archeologically intensively studied, but as more work is conducted in other regions, as more osteological and fossil data become available, either physically (in museums and collections) or digitally (as three-dimensional optical or CT scans), and as the various non-scientific hurdles related to accessing these data diminish, this type of investigation can be used to shed light on regional or larger-scale developments.

Finally, we are guilty of producing yet another study of vowel production here (although we do provide the formants F1 to F5 and the spectra up to 5000 Hz), but this can be extended to other speech sounds and vocal tract structures as well. Just to cite a few possibilities, one could investigate the alveolar ridge and its effects on click consonants [91,141], hard palate shape and ‘r’ [41], dentition/bite and labiodentals [94], or larynx position and, indeed, vowels [4].

Putting these extensions together, we could suggest studies that would, for example, look at the osteological and fossil record of sub-Saharan Africa preceding the relatively recent Bantu expansion [142,143], focusing on the alveolar ridge and aiming to understand the time-depth and geographical extensions of ‘click languages’. More precisely, while currently there are but a few languages that integrate click consonants in their phonological inventories, mostly in southern and eastern Africa (and some Bantu languages that have borrowed clicks), there are intriguing suggestions that they are but remnants of a once widespread use of phonemic clicks [144–146]. If the alveolar ridge shape and size indeed bias the articulation and acoustics of clicks [91,141], then we might be able to infer if past populations were biased in such a way as to favour phonological clicks or not. (Incidentally, we could apply the same inferences to the existing pygmy groups: while their pre-Bantu languages are lost, they may very well have used phonemic clicks as well.)

Another example might even transgress the origins of modern humans and try to infer features of Neanderthal speech: if the negative effects of the edge-to-edge bite on labiodentals inferred for recent hunter-gatherers [94] also hold deeper in time, we can infer that pre-12 000 years ago modern humans, archaic humans, Neanderthals (and even *H. erectus*) probably did not use ‘f’ and ‘v’ that much [147]. Likewise, we might use the shape of the Neanderthal hard palate to infer something about the vowels that their languages might have used, or even how they might have articulated their ‘r’s.

But we also need to know more about the type and patterning of variation in vocal tract anatomy, physiology and control in present-day humans, and its effects on speech and language, before we can generate informed hypotheses about the past. The work we presented here, concerning the hard palate [41,88], the alveolar ridge [91], and the bite [94], are (we hope) just the beginning of a vast research programme. Other directions could concern the curvature of the cervical spine and pitch [148,149], or the anatomy of the nasal cavity and the anatomy and physiology of the velum and vowel nasalization. While we do not know how widespread this variation is and how important its effects on speech and language are, the available evidence suggests that this is a worthwhile direction of future research. In the end, the breadth of these questions is only limited by our imagination, the fossil and osteological data, and the kind of relationships between vocal tract anatomy, sound change and linguistic diversity that future work will establish.

Such investigations assume that the processes, forces and mechanisms that we observe today (in particular, the effects of vocal tract anatomy on speech and language) were also at work in the past in pretty much the same way—what is known as the uniformitarian principle. However, it is currently unclear how this principle should be applied in practice, because the farther back in time we go the more different things (gradually) become, until we cross into the long evolutionary history preceding the emergence of language as we know it [9,150]. Even closer to the present, if the link between food, bite and labiodentals [94] holds, then strict uniformitarianism breaks down at the dawn of agriculture, as we cannot expect pre-12 000-years old languages to have the same distribution of labiodentals as present-day languages, but the uniformitarian principle would still largely apply, as the same articulatory constraints and affordances worked then as they do today. (For more nuanced discussions and further developments, see [16], as well as [151–153].)

## Data accessibility

The R code and data needed to reproduce the simulation plots and results reported here and in the electronic supplementary materials are freely available as described in the electronic supplementary materials. The 3D STL models of the four crania used in the simulations are available upon request from W.A.B.

## References

[1] Howard DM, Schofield J, Fletcher J, Baxter K, Iball GR, Buckley SA. 2020 Synthesis of a vocal sound from the 3,000 year old mummy, Nesyamun ‘True of Voice’. Sci. Rep. **10**, 1-6. (10.1038/s41598-019-56316-y) 31974412 PMC6978302

[2] Boë L-J, Heimb J-L, Hondac K, Maeda S. 2002 The potential Neandertal vowel space was as large as that of modern humans. J. Phon. **30**, 465-484. (10.1006/jpho.2002.0170)

[3] de Boer B, Fitch WT. 2010 Computer models of vocal tract evolution: an overview and critique. Adapt. Behav. **18**, 36-47. (10.1177/1059712309350972)

[4] Janssen R, Moisik SR, Dediu D. 2019 The effects of larynx height on vowel production are mitigated by the active control of articulators. J. Phon. **74**, 1-17. (10.1016/j.wocn.2019.02.002)

[5] Lieberman P, Crelin ES, Klatt DH. 1972 Phonetic ability and related anatomy of the newborn and adult human, Neanderthal man, and the chimpanzee. Am. Anthropol. **74**, 287-307. (10.1525/aa.1972.74.3.02a00020)

[6] Lieberman P, Crelin ES. 1971 On the speech of Neanderthal man. Linguist. Inq. **2**, 203-222.

[7] Berwick RC, Chomsky N. 2017 Why only us: recent questions and answers. J. Neurolinguistics 43(B), 166-177. (10.1016/j.jneuroling.2016.12.002)

[8] Hauser MD, Yang C, Berwick RC, Tattersall I, Ryan M, Watumull J, Chomsky N, Lewontin R. 2014 The mystery of language evolution. Front. Psychol. **5**, 401. (10.3389/fpsyg.2014.00401) 24847300 PMC4019876

[9] Dediu D, Levinson SC. 2013 On the antiquity of language: the reinterpretation of Neandertal linguistic capacities and its consequences. Front. Lang. Sci. **4**, 397. (10.3389/fpsyg.2013.00397) PMC3701805 23847571

[10] Dediu D, Levinson SC. 2018 Neanderthal language revisited: not only us. Curr. Opin. Behav. Sci. **21**, 49-55. (10.1016/j.cobeha.2018.01.001)

[11] Renfrew C, McMahon A, Trask L (eds). 2000 Time depth in historical linguistics. Cambridge, UK: McDonald Institute.

[12] Bowern C, Evans B. 2014 The Routledge handbook of historical linguistics. New York, NY: Routledge.

[13] Campbell L. 2004 Historical linguistics : an introduction. Edinburgh, UK: Edinburgh University Press.

[14] Lass R. 1997 Historical linguistics and language change. Cambridge, UK: Cambridge University Press.

[15] Mallory JP, Adams DQ. 2006 The Oxford introduction to proto-Indo-European and the proto-Indo-European world. Oxford, UK: Oxford University Press.

[16] Everett C. 2021 The sounds of prehistoric speech. Phil. Trans. R. Soc. B **376**, 20200195. (10.1098/rstb.2020.0195) 33745314 PMC8059574

[17] Renfrew C, Nettle D (eds). 1999 Nostratic: examining a linguistic macrofamily. Cambridge, UK: McDonald Institute.

[18] Salmons JC, Joseph BD (eds). 1998 Nostratic: sifting the evidence. Amsterdam, The Netherlands: John Benjamins.

[19] Greenberg JH. 2000 Indo-European and its closest relatives: the eurasiatic language family, volume 2, lexicon. Stanford, CA: Stanford University Press.

[20] Robbeets M, Bouckaert R. 2018 Bayesian phylolinguistics reveals the internal structure of the Transeurasian family. J. Lang. Evol. **3**, 145-162. (10.1093/jole/lzy007)

[21] Starostin SA, Dybo AV, Mudrak OA. 2003 Etymological dictionary of the Altaic languages. Leiden, The Netherlands: Brill.

[22] Heggarty P. 2013 Ultraconserved words and Eurasiatic? The ‘faces in the fire’ of language prehistory. Proc. Natl Acad. Sci. USA **110**, E3254. (10.1073/pnas.1309114110) 23918403 PMC3761561

[23] Barbieri C, Butthof A, Bostoen K, Pakendorf B. 2013 Genetic perspectives on the origin of clicks in Bantu languages from southwestern Zambia. Eur. J. Hum. Genet. **21**, 430-436. (10.1038/ejhg.2012.192) 22929022 PMC3598317

[24] Haak W et al. 2015 Massive migration from the steppe was a source for Indo-European languages in Europe. Nature **522**, 207-211. (10.1038/nature14317) 25731166 PMC5048219

[25] Olalde I et al. 2018 The Beaker phenomenon and the genomic transformation of northwest Europe. Nature **555**, 190-196. (10.1038/nature25738) 29466337 PMC5973796

[26] Heggarty P. 2010 Prehistory through language and archaeology. In The Routledge handbook of historical linguistics (eds C Bowern, B Evans), pp. 498-626. New York, NY: Routledge.

[27] Bouckaert R, Lemey P, Dunn M, Greenhill SJ, Alekseyenko AV, Drummond AJ, Gray RD, Suchard MA, Atkinson QD. 2012 Mapping the origins and expansion of the Indo-European language family. Science **337**, 957-960. (10.1126/science.1219669) 22923579 PMC4112997

[28] Bowern C, Atkinson Q. 2012 Computational phylogenetics and the internal structure of Pama-Nyungan. Language **88**, 817-845. (10.1353/lan.2012.0081)

[29] Gray RD, Atkinson QD. 2003 Language-tree divergence times support the Anatolian theory of Indo-European origin. Nature **426**, 435-439. (10.1038/nature02029) 14647380

[30] Honkola T, Vesakoski O, Korhonen K, Lehtinen J, Syrjänen K, Wahlberg N. 2013 Cultural and climatic changes shape the evolutionary history of the Uralic languages. J. Evol. Biol. **26**, 1244-1253. (10.1111/jeb.12107) 23675756

[31] Pagel M, Atkinson QD, Calude AS, Meade A. 2013 Ultraconserved words point to deep language ancestry across Eurasia. Proc. Natl Acad. Sci. USA **110**, 8471-8476. (10.1073/pnas.1218726110) 23650390 PMC3666749

[32] Wichmann S et al. 2012 *The ASJP Database (version 15)*.

[33] Jäger G. 2015 Support for linguistic macrofamilies from weighted sequence alignment. Proc. Natl Acad. Sci. USA **112**, 12 752-12 757. (10.1073/pnas.1500331112) PMC4611657 26403857

[34] Dediu D, Levinson SC. 2012 Abstract profiles of structural stability point to universal tendencies, family-specific factors, and ancient connections between languages. PLoS ONE **7**, e45198. (10.1371/journal.pone.0045198) 23028843 PMC3447929

[35] Atkinson QD. 2011 Phonemic diversity supports a serial founder effect model of language expansion from Africa. Science **332**, 346-349. (10.1126/science.1199295) 21493858

[36] Atkinson QD. 2012 Response to Comments on ‘Phonemic diversity supports a serial founder effect model of language expansion from Africa’. Science **335**, 657. (10.1126/science.1210005) 21493858

[37] Bowern C. 2011 Out of Africa? The logic of phoneme inventories and founder effects. Linguist. Typol. **15**, 207-216. (10.1515/lity.2011.015)

[38] Cysouw M, Dediu D, Moran S. 2012 Comment on ‘Phonemic diversity supports a serial founder effect model of language expansion from Africa’. Science **335**, 657. (10.1126/science.1208841) 22323803

[39] Dediu D, Janssen R, Moisik SR. 2017 Language is not isolated from its wider environment: vocal tract influences on the evolution of speech and language. Lang. Commun. **54**, 9-20. (10.1016/j.langcom.2016.10.002)

[40] Dediu D, Moisik SR. 2019 Biology matters: variation in vocal tract anatomy and language. Sel. Pap. Theor. Appl. Linguist. **23**, 19-33.

[41] Dediu D, Moisik SR. 2019 Pushes and pulls from below: anatomical variation, articulation and sound change. Glossa J. Gen. Linguist. **4**, 7. (10.5334/gjgl.646)

[42] The 1000 Genomes Project Consortium. 2015 A global reference for human genetic variation. Nature **526**, 68-74. (10.1038/nature15393) 26432245 PMC4750478

[43] Tracy TS et al*.* 2016 Interindividual variability in cytochrome P450-mediated drug metabolism. Drug Metab. Dispos. **44**, 343-351. (10.1124/dmd.115.067900) 26681736 PMC4767386

[44] Durand C, Rappold GA. 2013 Height matters—from monogenic disorders to normal variation. Nat. Rev. Endocrinol. **9**, 171-177. (10.1038/nrendo.2012.251) 23337954

[45] Furr RM, Bacharach VR. 2007 Psychometrics: an introduction, 1st edn. Thousand Oaks, CA: Sage Publications, Inc.

[46] Barbujani G, Colonna V. 2010 Human genome diversity: frequently asked questions. Trends Genet. **26**, 285-295. (10.1016/j.tig.2010.04.002) 20471132

[47] Jobling MA, Hollox E, Hurles M, Kivisild T, Tyler-Smith C. 2013 Human evolutionary genetics. New York, NY: Garland Science.

[48] Reich D. 2018 Who we are and how we got here: ancient DNA and the new science of the human past. Oxford, UK: Oxford University Press.

[49] Bowden R, MacFie TS, Myers S, Hellenthal G, Nerrienet E, Bontrop RE, Freeman C, Donnelly P, Mundy NI. 2012 Genomic tools for evolution and conservation in the chimpanzee: *Pan troglodytes* *ellioti* is a genetically distinct population. PLoS Genet. **8**, e1002504. (10.1371/journal.pgen.1002504) 22396655 PMC3291532

[50] Lewontin RC. 1972 The apportionment of human diversity. In Evolutionary biology (eds T Dobzhansky, MK Hecht, WC Steere), pp. 381-398. New York, NY: Springer.

[51] Novembre J et al*.* 2008 Genes mirror geography within Europe. Nature **456**, 98-101. (10.1038/nature07331) 18758442 PMC2735096

[52] Huxley J, Haddon AC. 1935 We Europeans: a survey of ‘racial’ problems. London, UK: J. Cape.

[53] Lippert-Rasmussen K. 2014 Born free and equal?: A philosophical inquiry into the nature of discrimination. Oxford, UK: Oxford University Press.

[54] Livingstone FB, Dobzhansky T. 1962 On the non-existence of human races. Curr. Anthropol. **3**, 279-281. (10.1086/200290)

[55] Roberts DE. 2011 Fatal invention: how science, politics, and big business re-create race in the twenty-first century. New York, NY: New Press.

[56] Saini A. 2019 Superior: the return of race science. London, UK: HarperCollins.

[57] Nerbonne J. 2009 Data-driven dialectology. Lang. Linguist. Compass **3**, 175-198. (10.1111/j.1749-818X.2008.00114.x)

[58] Meyerhoff M. 2015 Introducing sociolinguistics. New York, NY: Taylor & Francis.

[59] Allott R. 1994 Motor theory of language: the diversity of languages. In Studies in language origins (eds J Wind, A Jonker, R Allott, L Rolfe), pp. 125-160. Amsterdam, The Netherlands: John Benjamins Publishing Company.

[60] Brosnahan LF. 1961 The sounds of language: an inquiry into the role of genetic factors in the development of sound systems. Cambridge, UK: W. Heffer and Sons.

[61] Brunner J, Fuchs S, Perrier P. 2009 On the relationship between palate shape and articulatory behavior. J. Acoust. Soc. Am. **125**, 3936-3949. (10.1121/1.3125313) 19507976

[62] Lammert A, Proctor M, Narayanan S. 2013 Morphological variation in the adult hard palate and posterior pharyngeal wall. J. Speech Lang. Hear. Res. **56**, 521-530. (10.1044/1092-4388(2012/12-0059)) 23690566 PMC3885355

[63] Ohala JJ. 1989 Sound change is drawn from a pool of synchronic variation. In Language change: contributions to the study of its causes (eds LE Breivik, EH Jahr), pp. 173-198. Berlin, Germany: Mouton de Gruyter.

[64] Weirich M, Fuchs S. 2013 Palatal morphology can influence speaker-specific realizations of phonemic contrasts. J. Speech Lang. Hear. Res. **56**, 1894-1908. (10.1044/1092-4388(2013/12-0217)) 24687445

[65] Yu ACL. 2013 Origins of sound change: approaches to phonologization. Oxford, UK: Oxford University Press.

[66] Galluccio G, Castellano M, La Monaca C. 2012 Genetic basis of non-syndromic anomalies of human tooth number. Arch. Oral Biol. **57**, 918-930. (10.1016/j.archoralbio.2012.01.005) 22325622

[67] Jheon AH, Seidel K, Biehs B, Klein OD. 2013 From molecules to mastication: the development and evolution of teeth. Wiley Interdiscip. Rev. Dev. Biol. **2**, 165-182. (10.1002/wdev.63) 24009032 PMC3632217

[68] Dixon MJ, Marazita ML, Beaty TH, Murray JC. 2011 Cleft lip and palate: synthesizing genetic and environmental influences. Nat. Rev. Genet. **12**, 167-178. (10.1038/nrg2933) 21331089 PMC3086810

[69] Leslie EJ, Marazita ML. 2013 Genetics of cleft lip and cleft palate. Am. J. Med. Genet. C **163**, 246-258. (10.1002/ajmg.c.31381) PMC3925974 24124047

[70] Gokhman D et al*.* 2020 Differential DNA methylation of vocal and facial anatomy genes in modern humans. Nat. Commun. **11**, 1-21. (10.1038/s41467-020-15020-6) 32132541 PMC7055320

[71] Murphy WA, zur Nedden D, Gostner P, Knapp R, Recheis W, Seidler H. 2003 The iceman: discovery and imaging. Radiology **226**, 614-629. (10.1148/radiol.2263020338) 12601185

[72] Van der Sanden W. 1996 Through nature to eternity: the bog bodies of northwest Europe. Amsterdam, The Netherlands: Batavian Lion. See https://books.google.fr/books/about/Through_Nature_to_Eternity.html?id=GgFsQgAACAAJ&redir_esc=y.

[73] Everett C, Blasi DE, Roberts SG. 2015 Climate, vocal folds, and tonal languages: connecting the physiological and geographic dots. Proc. Natl Acad. Sci. USA **112**, 1322-1327. (10.1073/pnas.1417413112) 25605876 PMC4321236

[74] Everett C. 2017 Languages in drier climates use fewer vowels. Front. Psychol. **8**, 1285. (10.3389/fpsyg.2017.01285) 28798711 PMC5529419

[75] Bastir M, Godoy P, Rosas A. 2011 Common features of sexual dimorphism in the cranial airways of different human populations. Am. J. Phys. Anthropol. **146**, 414-422. (10.1002/ajpa.21596) 21994017

[76] Hall RL. 2005 Energetics of nose and mouth breathing, body size, body composition, and nose volume in young adult males and females. Am. J. Hum. Biol. **17**, 321-330. (10.1002/ajhb.20122) 15849711

[77] Moreddu E, Puymerail L, Michel J, Achache M, Dessi P, Adalian P. 2013 Morphometric measurements and sexual dimorphism of the piriform aperture in adults. Surg. Radiol. Anat. **35**, 917-924. (10.1007/s00276-013-1116-2) 23625070

[78] Bastir M, Rosas A. 2013 Cranial airways and the integration between the inner and outer facial skeleton in humans. Am. J. Phys. Anthropol. **152**, 287-293. (10.1002/ajpa.22359) 23999909

[79] Roseman CC, Weaver TD. 2004 Multivariate apportionment of global human craniometric diversity. Am. J. Phys. Anthropol. **125**, 257-263. (10.1002/ajpa.10424) 15386236

[80] Noback ML, Harvati K, Spoor F. 2011 Climate-related variation of the human nasal cavity. Am. J. Phys. Anthropol. **145**, 599-614. (10.1002/ajpa.21523) 21660932

[81] Lieberman P. 1994 Functional tongues and Neanderthal vocal tract reconstruction: a reply to Dr. Houghton (1993). Am. J. Phys. Anthropol. **95**, 443-450; discussion 450–2. (10.1002/ajpa.1330950408) 7864065

[82] Lieberman P. 2016 The evolution of language and thought. J. Anthropol. Sci. **94**, 127-146. (10.4436/JASS.94029) 26963222

[83] Fitch WT. 2000 The phonetic potential of nonhuman vocal tracts: comparative cineradiographic observations of vocalizing animals. Phonetica **57**, 205-218. (10.1159/000028474) 10992141

[84] Fitch WT, Reby D. 2001 The descended larynx is not uniquely human. Proc. R. Soc. Lond. B **268**, 1669-1675. (10.1098/rspb.2001.1704) PMC1088793 11506679

[85] D'Anastasio R et al*.* 2013 Micro-biomechanics of the Kebara 2 hyoid and its implications for speech in Neanderthals. PLoS ONE **8**, e82261. (10.1371/journal.pone.0082261) 24367509 PMC3867335

[86] Brunner J, Fuchs S, Perrier P. 2005 The influence of the palate shape on articulatory token-to-token variability. ZAS Pap. Linguist. **42**, 43-67. (10.21248/zaspil.42.2005.273)

[87] Birkholz P. 2013 Modeling consonant-vowel coarticulation for articulatory speech synthesis. PLoS ONE **8**, e60603. (10.1371/journal.pone.0060603) 23613734 PMC3628899

[88] Dediu D, Janssen R, Moisik SR. 2019 Weak biases emerging from vocal tract anatomy shape the repeated transmission of vowels. *Nat**.* Hum. Behav. **3**, 1107-1115. (10.1038/s41562-019-0663-x) 31427785

[89] Janssen R, Moisik SR, Dediu D. 2018 Modelling human hard palate shape with Bézier curves. PLoS ONE **13**, e0191557. (10.1371/journal.pone.0191557) 29447175 PMC5813942

[90] Lloyd JE, Stavness I, Fels S. 2012 ArtiSynth: a fast interactive biomechanical modeling toolkit combining multibody and finite element simulation. In Soft tissue biomechanical modeling for computer assisted surgery (ed. Y Payan), pp. 355-394. Berlin, Germany: Springer.

[91] Moisik SR, Dediu D. 2017 Anatomical biasing and clicks: evidence from biomechanical modeling. J. Lang. Evol. **2**, 37-51. (10.1093/jole/lzx004)

[92] Mielke J, Smith B, Fox MJ. 2017 Implications of covert articulatory variation for several phonetic variables in Raleigh, North Carolina English. J. Acoust. Soc. Am. **141**, 3981. (10.1121/1.4989092)

[93] Smith BJ, Mielke J, Magloughlin L, Wilbanks E. 2019 Sound change and coarticulatory variability involving English /ɹ/. Glossa J. Gen. Linguist. **4**, 63. (10.5334/gjgl.650)

[94] Blasi DE, Moran S, Moisik SR, Widmer P, Dediu D, Bickel B. 2019 Human sound systems are shaped by post-Neolithic changes in bite configuration. Science **363**, eaav3218. (10.1126/science.aav3218) 30872490

[95] Deter CA. 2009 Gradients of occlusal wear in hunter-gatherers and agriculturalists. Am. J. Phys. Anthropol. **138**, 247-254. (10.1002/ajpa.20922) 18773466

[96] Kaifu Y. 2000 Tooth wear and compensatory modification of the anterior dentoalveolar complex in humans. Am. J. Phys. Anthropol. **111**, 369-392. (10.1002/(SICI)1096-8644(200003)111:3<369::AID-AJPA6>3.0.CO;2-#) 10685038

[97] Kaifu Y, Kasai K, Townsend GC, Richards LC. 2003 Tooth wear and the ‘design’ of the human dentition: a perspective from evolutionary medicine. Am. J. Phys. Anthropol. **122**, 47-61. (10.1002/ajpa.10329) 14666533

[98] von Cramon-Taubadel N. 2011 Global human mandibular variation reflects differences in agricultural and hunter-gatherer subsistence strategies. Proc. Natl Acad. Sci. USA **108**, 19 546-19 551. (10.1073/pnas.1113050108) PMC3241821 22106280

[99] Alemseged Z, Spoor F, Kimbel WH, Bobe R, Geraads D, Reed D, Wynn JG. 2006 A juvenile early hominin skeleton from Dikika, Ethiopia. Nature **443**, 296-301. (10.1038/nature05047) 16988704

[100] Arensburg B, Tillier AM, Vandermeersch B, Duday H, Schepartz LA, Rak Y. 1989 A Middle Palaeolithic human hyoid bone. Nature **338**, 758-760. (10.1038/338758a0) 2716823

[101] Martínez I, Arsuaga JL, Quam R, Carretero JM, Gracia A, Rodríguez L. 2008 Human hyoid bones from the middle Pleistocene site of the Sima de los Huesos (Sierra de Atapuerca, Spain). J. Hum. Evol. **54**, 118-124. (10.1016/j.jhevol.2007.07.006) 17804038

[102] Capasso L, Michetti E, D'Anastasio R. 2008 A *Homo erectus* hyoid bone: possible implications for the origin of the human capability for speech. Coll. Antropol. **32**, 1007-1011. 19149203

[103] Capasso L, D'Anastasio R, Mancini L. 2016 New evaluation of the Castel di Guido ‘hyoid’. J. Anthropol. Sci. **94**, 231-235. (10.4436/JASS.94021) 26656957

[104] Frayer DW. 2017 Talking hyoids and talking Neanderthals. In Human paleontology and prehistory (eds A Marom, E Hovers), pp. 233-237. Cham, Switzerland: Springer. (10.1007/978-3-319-46646-0_17)

[105] Beals ME, Frayer DW, Radovčić J, Hill CA. 2016 Cochlear labyrinth volume in Krapina Neandertals. J. Hum. Evol. **90**, 176-182. (10.1016/j.jhevol.2015.09.005) 26603101

[106] Martínez I et al. 2004 Auditory capacities in Middle Pleistocene humans from the Sierra de Atapuerca in Spain. Proc. Natl Acad. Sci. USA **101**, 9976-9981. (10.1073/pnas.0403595101) 15213327 PMC454200

[107] Martínez I, Quam RM, Rosa M, Jarabo P, Lorenzo C, Arsuaga JL. 2008 Auditory capacities of human fossils: a new approach to the origin of speech. J. Acoust. Soc. Am. **123**, 3606. (10.1121/1.2934784)

[108] Stoessel A, David R, Gunz P, Schmidt T, Spoor F, Hublin J-J. 2016 Morphology and function of Neandertal and modern human ear ossicles. Proc. Natl Acad. Sci. USA **113**, 11 489-11 494. (10.1073/pnas.1605881113) PMC5068335 27671643

[109] Beaudet A. 2017 The emergence of language in the hominin lineage: perspectives from fossil endocasts. Front. Hum. Neurosci. **11**, 427. (10.3389/fnhum.2017.00427) 28878641 PMC5572361

[110] Holloway RL. 2009 Brain fossils: endocasts. Encycl. Neurosci. **2**, 253-261.

[111] DeGusta D, Gilbert WH, Turner SP. 1999 Hypoglossal canal size and hominid speech. Proc. Natl Acad. Sci. USA **96**, 1800-1804. (10.1073/pnas.96.4.1800) 9990105 PMC15600

[112] Jungers WL, Pokempner AA, Kay RF, Cartmill M. 2003 Hypoglossal canal size in living hominoids and the evolution of human speech. Hum. Biol. **75**, 473-484. (10.1353/hub.2003.0057) 14655872

[113] Galloway A, Willey P, Snyder L. 1997 Human bone mineral densities and survival of bone elements: a contemporary sample. In Forensic taphonomy: the postmortem fate of human remains (eds WD Haglund, MH Sorg), pp. 295-317. Boca Raton, FL: CRC Press.

[114] Waldron T. 1987 The relative survival of the human skeleton: implications for paleopathology. In Death, decay and reconstruction (eds A Boddington, AN Garland, RC Janaway), pp. 55-64. Manchester, UK: Manchester University Press.

[115] Baetsen WA. 2016 Geometric morphometric analysis of the ‘skeletal vocal tract’: a first step in involving osteoarchaeology in the search for a potential ‘genetic bias’ for language, using two Dutch historical skeletal populations. MSc thesis, University of Leiden, Leiden, The Netherlands. (https://hdl.handle.net/1887/45959)

[116] Bosman AM. 2016 Talking heads. MSc thesis, Human Osteology and Funerary Archaeology, University of Leiden, Leiden, The Netherlands.

[117] Bosman AM, Moisik SR, Dediu D, Waters-Rist A. 2017 Talking heads: morphological variation in the human mandible over the last 500 years in the Netherlands. HOMO J. Comp. Hum. Biol. **68**, 329-342. (10.1016/j.jchb.2017.08.002) 28987534

[118] Hoppa RD. 2000 Population variation in osteological aging criteria: an example from the pubic symphysis. Am. J. Phys. Anthropol. **111**, 185-191. (10.1002/(SICI)1096-8644(200002)111:2<185::AID-AJPA5>3.0.CO;2-4) 10640946

[119] Konigsberg LW, Hens SM. 1998 Use of ordinal categorical variables in skeletal assessment of sex from the cranium. Am. J. Phys. Anthropol. **107**, 97-112. (10.1002/(SICI)1096-8644(199809)107:1<97::AID-AJPA8>3.0.CO;2-A) 9740304

[120] Ubelaker DH, Khosrowshahi H. 2019 Estimation of age in forensic anthropology: historical perspective and recent methodological advances. Forensic Sci. Res. **4**, 1-9. (10.1080/20961790.2018.1549711) 30915413 PMC6427487

[121] Cignoni P, Callieri M, Corsini M, Dellepiane M, Ganovelli F, Ranzuglia G. 2008 MeshLab: an open-source mesh processing tool. In Eurographics Italian chapter conference (eds V Scarano, RD Chiara, U Erra). The Eurographics Association.

[122] Wiley DF et al. 2005 Evolutionary morphing. In Conf. Proc. VIS 05. IEEE visualization, 23–28 October, Minneapolis, MN, pp. 431-438. New York, NY: IEEE.

[123] Zelditch ML, Swiderski DL, Sheets HD. 2012 Geometric morphometrics for biologists: a primer. Amsterdam, The Netherlands: Academic Press.

[124] Brachin P. 1985 The Dutch language: a survey. Leiden, The Netherlands: EJ Brill Publishing Company.

[125] De Vin A. 1952 Het dialect van Schouwen-Duiveland. Assen, The Netherlands: Van Gorcum & Comp. N.V.

[126] Janssens G, Marynissen A. 2005 Het Nederlands van vroeger en nu. Leuven, The Netherlands: Uitgeverij Acco.

[127] Van den Toorn MC, Pijnenburg WJJ, van Leuvenstijn JA, van der Horst JM. 1997 Geschiedenis van de Nederlandse taal. Amsterdam, The Netherlands: Amsterdam University Press.

[128] Katzenberg MA, Waters-Rist AL. 2018 Stable isotope analysis. In Biological anthropology of the human skeleton (eds MA Katzenberg, AL Grauer), pp. 467-504. New York, NY: John Wiley & Sons.

[129] Henry AG, Piperno DR. 2008 Using plant microfossils from dental calculus to recover human diet: a case study from Tell al-Raqā’i, Syria. J. Archaeol. Sci. **35**, 1943-1950. (10.1016/j.jas.2007.12.005)

[130] Weyrich LS et al*.* 2017 Neanderthal behaviour, diet, and disease inferred from ancient DNA in dental calculus. Nature **544**, 357-361. (10.1038/nature21674) 28273061

[131] Richards MP, Trinkaus E. 2009 Out of Africa: modern human origins special feature: isotopic evidence for the diets of European Neanderthals and early modern humans. Proc. Natl Acad. Sci. USA **106**, 16 034-16 039. (10.1073/pnas.0903821106) PMC2752538 19706482

[132] Wißing C et al*.* 2019 Stable isotopes reveal patterns of diet and mobility in the last Neandertals and first modern humans in Europe. Sci. Rep. **9**, 4433. (10.1038/s41598-019-41033-3) 30872714 PMC6418202

[133] Mahoney P. 2006 Dental microwear from Natufian hunter-gatherers and early Neolithic farmers: comparisons within and between samples. Am. J. Phys. Anthropol. **130**, 308-319. (10.1002/ajpa.20311) 16395722

[134] Smith BH. 1984 Patterns of molar wear in hunter-gatherers and agriculturalists. Am. J. Phys. Anthropol. **63**, 39-56. (10.1002/ajpa.1330630107) 6422767

[135] Schats R. 2016 Life in transition: an osteoarchaeological perspective of the consequences of mediëval socioeconomic developments in Holland and Zeeland (AD 1000–1600). Doctoral thesis, University of Leiden, Leiden, The Netherlands. See https://openaccess.leidenuniv.nl/handle/1887/43926.

[136] van Hattum IJ. 2015 ‘What's on the Menu?’: Diet in Medieval Holland: a stable carbon and nitrogen isotope analysis of bone ‘collagen’ from early medieval Blokhuizen and late medieval Alkmaar. MSc thesis, University of Leiden, Leiden, The Netherlands. (https://hdl.handle.net/1887/35568)

[137] Waters-Rist AL, Hoogland MLP. 2018 The role of infant feeding and childhood diet in vitamin D deficiency in a nineteenth-century rural Dutch community. Bioarchaeol. Int. **2**, 95-116. (10.5744/bi.2018.1020)

[138] Beck JM. 1988 Organic variation and voice quality. PhD thesis, University of Edinburgh, UK. See https://era.ed.ac.uk/handle/1842/7125.

[139] Laver J. 1980 The phonetic description of voice quality. Cambridge, UK: Cambridge University Press.

[140] Richter D et al*.* 2017 The age of the hominin fossils from Jebel Irhoud, Morocco, and the origins of the Middle Stone Age. Nature **546**, 293-296. (10.1038/nature22335) 28593967

[141] Moisik SR, Dediu D. 2020 The ArtiVarK click study: documenting click production and substitution strategies by learners in a large phonetic training and vocal tract imaging study. In The handbook of clicks (ed B Sands), pp. 384-417. Leiden, The Netherlands: Brill.

[142] de Filippo C, Bostoen K, Stoneking M, Pakendorf B. 2012 Bringing together linguistic and genetic evidence to test the Bantu expansion. Proc. R. Soc. B **279**, 3256-3263. (10.1098/rspb.2012.0318) PMC3385717 22628476

[143] Grollemund R, Branford S, Bostoen K, Meade A, Venditti C, Pagel M. 2015 Bantu expansion shows that habitat alters the route and pace of human dispersals. Proc. Natl Acad. Sci. USA **112**, 13 296-13 301. (10.1073/pnas.1503793112) PMC4629331 26371302

[144] Engstrand O. 1997 Why are clicks so exclusive. In Proc. Fonetik-97, Umeå University, PHONUM **4**, 191-194.

[145] Fleming L. 2017 Phoneme inventory size and the transition from monoplanar to dually patterned speech. J. Lang. Evol. **2**, 52-66. (10.1093/jole/lzx010)

[146] Traunmüller H. 2003 Clicks and the idea of a human protolanguage. PHONUM **9**, 1-4.

[147] Spitzer M. 2019 Neandertaler ohne F. Nervenheilkunde **36**, 578-581. (10.1055/a-0916-1232)

[148] Honda K, Hirai H, Masaki S, Shimada Y. 1999 Role of vertical larynx movement and cervical lordosis in F0 control. Lang. Speech **42**, 401-411. (10.1177/00238309990420040301) 10845244

[149] Moisik SR, Yun DPZ, Dediu D. 2019 Active adjustment of the cervical spine during pitch production compensates for shape: the ArtiVarK study. In Proc. 19th Int. Congress of Phonetic Sciences (ICPhS 2019), 5–9 August 2019, Melbourne, Australia, p. 5. Canberra, Australia: Australasian Speech Science and Technology Association Inc.

[150] Fitch WT. 2010 The evolution of language. Cambridge, UK: Cambridge University Press.

[151] Christy TC. 1983 Uniformitarianism in linguistics. Amsterdam, The Netherlands: John Benjamins Publishing Company. See https://benjamins.com/catalog/sihols.31.

[152] Walkden G. 2019 The many faces of uniformitarianism in linguistics. Glossa J. Gen. Linguist. **4**, 52. (10.5334/gjgl.888)

[153] Trudgill P. 2020 Millennia of language change: sociolinguistic studies in deep historical linguistics. Cambridge, UK: Cambridge University Press.

